# Chemical constituents and coagulation activity of *Syringa oblata* Lindl flowers

**DOI:** 10.1186/s13065-019-0621-8

**Published:** 2019-08-14

**Authors:** Lili Cui, Miyun Hu, Pengran Cao, Yun Niu, Changqin Li, Zhenhua Liu, Wenyi Kang

**Affiliations:** 1 National R& D Center for Edible Fungus Processing Technology, Henan University, Kaifeng, 475004 China; 2 Joint International Research Laboratory of Food & Medicine Resource Function, Kaifeng, Henan Province 475004 China; 3 Kaifeng Key Laboratory of Functional Components in Health Food, Kaifeng, 475004 China

**Keywords:** *Syringa oblata* Lindl flowers, Chemical constituents, Coagulation activity

## Abstract

The leaves and bark of *Syringa oblata* Lindl are used as folk medicine which has heat-clearing, detoxifying, dampness-removing and jaundice-relieving effects. There are many studies about leaves of *S. oblata* because of its abundant resource, however, less reports about the components of *S. oblata* flowers. The previous studies on *S. oblate* flowers were mainly focused on the volatile components and its traditional pharmacological activity. Thus, this study aimed to investigate the nonvolatile chemical constituents and the coagulation activity of *S. oblate* flowers. The chemical constituents of *S. oblate* flowers were isolated with various column chromatographies and coagulation activity of the major constituents was investigated by assaying the activated partial thromboplastin time (**APTT**), prothrombin time (**PT**), thrombin time (**TT**) and fibrinogen (**FIB**) on plasma of rabbit in vitro. Fifteen known compounds (namely compound **1**-**15**) were isolated from *S. oblata* flowers. Compound **6**, **10**, **11** and **14** were isolated from *Syringa* genus for the first time. Compound **1**, **2**, **4**, **5**, **8** and **9** were isolated from the plant for the first time. The results of coagulation activity showed that water part of *S. oblate* flowers, lauric acid and kaempferol-rutinose significantly shorten PT (*P *< 0.001), TT (*P *< 0.001) and APTT (*P *< 0.001) compared with blank group, thus revealed that water extract of *S. oblate* flowers, lauric acid and kaempferol-rutinose possessed the procoagulant activity, but the effects were not better than that of Yunnan Baiyao as positive control.

## Introduction

*Syringa oblata* Lindl, a medicinal plant which has the characteristics of trees or shrubs of the Oleaceae family, is native to north China. *S. oblate* tastes bitter, and has quality of cold. Chinese Materia Medica records that the leaves and bark of *S. oblata* have been used as folk medicine, which have heat-clearing, detoxifying, dampness -removing and jaundice-relieving effect [1].

Many studies were reported on the chemical constituents of *S. oblate* in China. Zhang et al. [2–6] isolated more than 50 compounds from the twigs, bark, leaves, alabastrum, seeds, and seed crust of *S. oblate*. These compounds were identified as oleanolic acid, lupinic acid, lupeol, 4-hydroxyphenethyl alcohol, 3,4-dihydroxyphenylethanol, *p*-hydroxyphenylethanol acetate, 2-(3,4-dihydroxy) phenyl ethyl acetate, *p*-hydroxyphenylethyl propyl ester, (8E)-ligstroside, oleuropein, syringopicroside, lariciresinol and esculetin, respectively. Tian et al. [7] isolated 9 compounds, including (+) pinoresinol-4″-*O*-*β*-d-glucopyranoside, (+)lariciresinol-4-*O*-*β*-d-glucoside, and epipinoresinol-4-*O*-*β*-d-glucopyranoside, from the leaves of *S. oblate*. Zhou [8] reported 2-furancarboxylic, mannitol, cyclohexane-1,2,3,4,5,6- hexaol, succinic acid, *p*-hydroxyphenylethyl alcohol and formononetin isolated from the leaves of *S. oblate*. Yang et al. [9] analyzed the compositions in the essential oil from fruits and leaves of *S. oblate*. Jiao et al. [10] found that the main component of the dried flowers of *S. oblate* were the same as those in the fruits and leaves.

These references indicate that triterpenes, phenethyl alcohol, phenylpropanoid and iridoid compounds are the main components in *S. oblate*. However, the flavonoids, organic acids and other constituents have been less reported.

In view of the fact that the *S. oblate* has a wide range of biological activities, including antibacterial, anti-inflammatory, antiviral, anti-tussive and expectorant effect, liver protection and cholagogue etc. [11]. The previous studies on *S. oblate* flowers were mainly focused on the volatile components and its traditional pharmacological activity. Thus, this study aimed to investigate the nonvolatile chemical constituents and the coagulation activity of *S. oblate* flowers.

## Methods

### Chemicals and material

The chemicals and material were similar to our previous research [12].

### Plant material

*Syringa oblata* flowers were collected in April 2015 from the Kaifeng region of Henan Province, China and identified by Professor Changqin Li. A voucher specimen was deposited in National R & D Center for Edible Fungus Processing Technology, Henan University.

### Animal

The male rabbit (approximately 20 months old, weight from 2.0 to 2.5 kg) was provided by Kaifeng Key Laboratory of Functional Components in Health Food (2016-02) to evaluate anticoagulant effect in vitro.

### Ethics information

The study obtained ethical clearance from the Ethics Committee of College of Medical, Henan University (NO: 2016-36). The rabbits were treated as per the guidelines on the care and use of animals for scientific purposes.

### Extraction and isolation

The extracted method was similar to our previous research [12]. The air-dried flowers of *S. oblata* (1.4 kg) were extracted with 70% ethanol to yield the crude extract (So. TE 378 g). The extract (378 g) was dissolved in MeOH-H_2_O (v:v = 3:1, 500 mL), and then mixed with D101 macroporous adsorbent resin. TE was separated by macroporous resin column chromatography, eluted with 20%, 40%, 60%, and 90% ethanol. After evaporation of the solvent, 235 g of water part, 27 g of 20% ethanol part, 61 g of 40% ethanol part, 20 g of 60% ethanol part and 35 g of 90% ethanol part were obtained.

The 60% ethanol part was separated on a silica gel H column by medium pressure liquid chromatography (MPLC), eluted with dichloromethane-methanol (v:v = 1:0–2:1) to obtain 2 fractions (F_1_–F_2_) based on TLC analyses. F_1_ was separated on a silica gel H column by MPLC, eluted with dichloromethane-acetone (v:v = 1:0–0:1) and then separated by Sephadex LH-20 (methanol) to obtain compound **1** (3 mg). F_2_ was separated with Sephadex LH-20 (methanol) and Sephadex LH-20 (methanol/water, 3:1, v/v) to obtain compound **2** (18 mg).

40% ethanol part was separated on a silica gel H column by MPLC, eluted with dichloromethane-methanol (v:v = 50:1–1:1) to obtain 6 fractions (P_1_–P_7_) based on TLC analyses. P_1_ was separated with Sephadex LH-20 (dichloromethane/methanol, 1:1, v/v) and Sephadex LH-20 (methanol) to obtain P_1-a_ and P_1-b_. F_1-a_ was subjected to atmospheric pressure chromatographic column of silica gel H with CHCl_2_-acetone (v:v = 1:0–0:1) to obtain compound **3** (20 mg). Compound **4** (16 mg) was obtained by the same separation method from F_1-b_. P_2_ was subjected to ordinary pressure chromatographic columns of silica gel H with dichloromethane-methanol (v:v = 80:1–15:1), and then separated with Sephadex LH-20 (methanol) to obtain compound **5** (4 mg). P_3_ was separated with Sephadex LH-20 (dichloromethane/methanol, 1:1, v/v) and Sephadex LH-20 (methanol), and then subjected to atmospheric pressure chromatographic column of silica gel H with dichloromethane _2_-acetone- methanol (v:v:v = 50:25:1) to obtain compound **6** (30 mg). P_4_ was separated on a silica gel H column by MPLC, eluted with dichloromethane-methanol (v:v = 100:1–5:1), and then separated with Sephadex LH-20 (methanol) to obtain compound **7** (11 mg). P_5_ was separated on a silica gel H column by MPLC, eluted with dichloromethane-methanol (v:v = 20:1–1:1), and then separated with Sephadex LH-20 (methanol) to obtain compound **8** (5 mg). P_6_ was separated with Sephadex LH-20 (dichloromethane/methanol, 1:1, v/v) and Sephadex LH-20 (methanol) to obtain compound **2** (74 mg). P_7_ was subjected to atmospheric pressure chromatographic column of silica gel H with dichloromethane-acetone-MeOH (v:v:v = 1:1:0.1–1:1:0.2) and then separated with Sephadex LH-20 (MeOH) to obtain compound **9** (39 mg).

The 90% ethanol part was separated by MPLC that was filled with silica gel H, eluted with petroleum ether-ethyl acetate (100:1–2:1, v/v) and dichloromethane- methanol (50:1–5:1, v/v) to obtain S_1_–S_5_. S_1_ was subjected to atmospheric pressure chromatographic column of silica gel H with petroleum ether-ethyl acetate-acetone (v:v:v = 100:1:1–2:1:1) and petroleum ether-ethyl acetate (v:v = 100:1–5:1) to obtain compound **10** (86 mg). S_2_ was subjected to decompressed chromatographic column of silica gel H with petroleum ether-ethyl acetate (v:v = 50:1–5:1), and then subjected to atmospheric pressure chromatographic column of silica gel H with petroleum ether- dichloromethane (v:v = 1:1–0:1) and petroleum ether-ethyl acetate (v:v = 20:1) to obtain compound **11** (25 mg). S_3_ was subjected to atmospheric pressure chromatographic column of silica gel H with petroleum ether- dichloromethane (v:v = 2:1–0:1) and dichloromethane-methanol (v:v = 20:3–10:1) to merge the same components based on TLC analysis. This part was then subjected to atmospheric pressure chromatographic column of silica gel H with petroleum ether-ethyl acetate (v:v = 20:3) to obtain compound **12** (45 mg). S_4_ was recrystallized to obtain white un-dissolved substance and yellow dissolved substance. The white un-dissolved substance was subjected to atmospheric pressure chromatographic column of silica gel H with dichloromethane-methanol (v:v = 50:1–3:1) to obtain compound **13** (39 mg). The yellow substance was separated with Sephadex LH-20 (dichloromethane/methanol, 1:1, v/v) to afford compound **14** (16 mg). S_5_ was recrystallized to obtain compound **15** (13 mg).

### The coagulation activity of *Syringa oblata* Lindl flowers in vitro

Blood samples were drawn from Rabbit’s Auricular vein without anaesthesia. The method was similar to our previous research [12]. APTT, PT,TT and FIB were determined.

For all the tests mentioned above, blank solvent (dimethyl sulphoxide: Tween 80: normal saline = 2:1:17) was used as negative control, while the drugs of breviscapine (13.3 mg/mL) and Yunnan baiyao (5 mg/mL) used in the clinics were used as positive control. All the samples were dissolved in blank solvent. The concentrations of compounds were 5 mg/mL and all the extract samples were 15 mg/mL. PT, APTT, TT and FIB tests were conducted with Semi-Automated Coagulation Analyzer (CPC Diagnostics Pvt. Ltd, India).

### Statistical analysis

The results of coagulation activity were expressed as mean ± standard deviation. The data analysis was performed by SPSS19.0 software with single factor analysis of variance (ANOVA One-Way) to determine the significant difference. The difference between groups with *P *< 0.05 and *P *< 0.001 were regarded as significant and highly significant, respectively. Results were shown in Table 1.

**Table 1:** The effects of *S. oblata* extract and compounds on APTT, PT, TT, and FIB in vitro ($\bar{x} \pm s$)

Groups	PT(s)	APTT(s)	TT(s)	FIB(g/L)
Blank	11.58 ± 0.26	20.03 ± 0.24	17.47 ± 0.36	3.16 ± 0.035
Breviscapine	14.90 ± 0.23***	23.68 ± 0.38***	20.05 ± 0.19***	2.97 ± 0.044**
Yunnan Baiyao	10.65 ± 0.38***	11.35 ± 0.94***	12.68 ± 0.13***	5.00 ± 0.14***
Water part	10.58 ± 0.22***	15.43 ± 0.22***^&&&^	16.43 ± 0.51***^&&&^	3.68 ± 0.087***^&&&^
20% ethanol part	11.83 ± 0.35	8.8 ± 0.32***^&&&^	16.43 ± 0.26***^&&&^	3.50 ± 0.11***^&&&^
40% ethanol part	12.35 ± 0.37***^###^	14.53 ± 0.66***^&&&^	16.45 ± 0.10***^&&&^	4.51 ± 0.077***^&&&^
60% ethanol part	NT	NT	NT	NT
90% ethanol part	12.03 ± 0.28**^###^	18.85 ± 0.26**^&&&^	16.75 ± 0.30**^&&&^	4.00 ± 0.062***^&&&^
So.TE	12.63 ± 0.30***^###^	14.58 ± 0.19***^&&&^	15.95 ± 0.21***^&&&^	3.78 ± 0.10***^&&&^
Lauric acid	10.85 ± 0.26***	18.35 ± 0.26***^&&&^	15.57 ± 0.34***^&&&^	3.95 ± 0.033***^&&&^
Dictamnoside A	11.50 ± 0.26	19.82 ± 0.59	13.93 ± 0.78***^&&&^	3.12 ± 0.050
Kaempferol-rutinose	10.55 ± 0.21^***^	17.45 ± 0.25***^&&&^	16.47 ± 0.29***^&&&^	3.22 ± 0.24

Results were expressed as mean ± SD, *n *= 4, *NT*: not detected

Compared with blank: *** *P *< 0.001; 0.001 < ** *P *< 0.01

Compared with breviscapine: ^###^ *P *< 0.001

Compared with Yunnan Baiyao: ^&&&^ *P *< 0.001

## Results

### Chemical constituents in *S. oblate* flowers

**Fig. 1:**
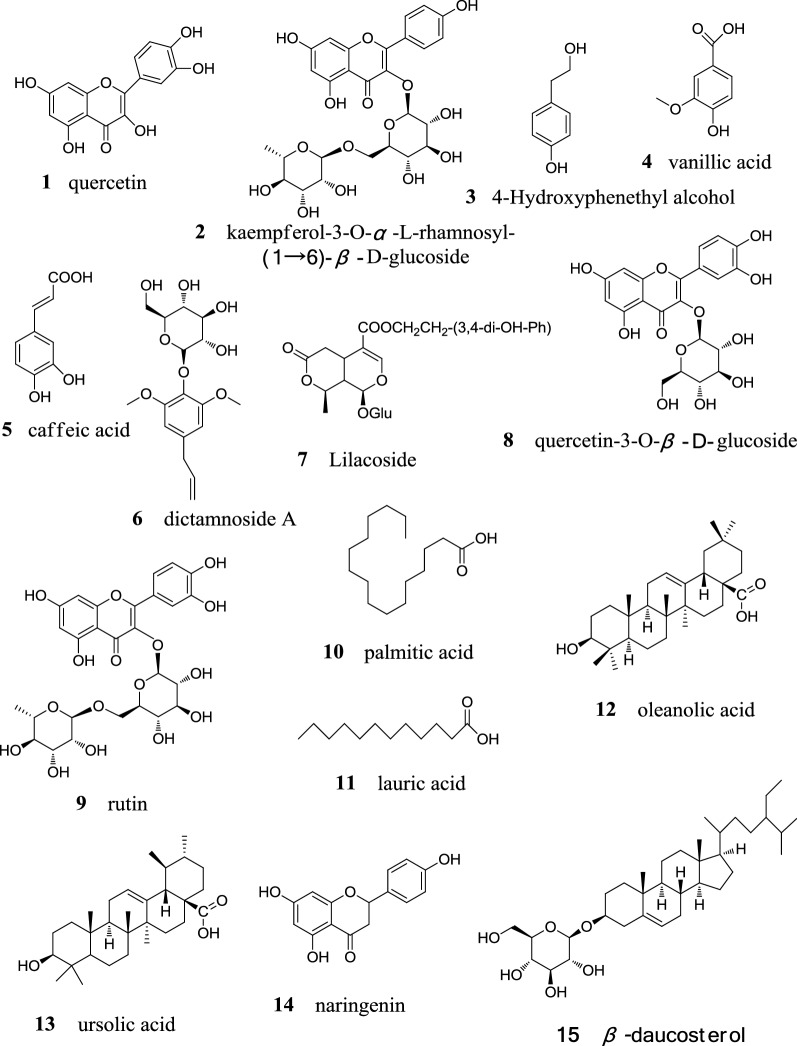
Structures of compound **1**–**15**

Fifteen known compounds (**1**–**15**) were isolated and identified from *S. oblata* flowers. The structures of compounds were shown in Fig. 1.

#### Compound 1

Yellow power. The molecular formula was C_15_H_10_O_7_. EI-MS *m/z*: 302[M]^+^. ^1^H-NMR (400 MHz, DMSO-*d*_6_) *δ*: 12.49 (1H, s, 5-OH), 7.67 (1H, s, H-2′), 7. 55 (1H, d, *J *= 8.0 Hz, H-6′), 6.89 (1H, d, *J *= 8.0 Hz, H-5′), 6.40 (1H, s, H-8), 6.18 (1H, s, H-6); ^13^C-NMR (100 MHz, DMSO-*d*_6_) *δ*: 146.74 (C-2), 135.66 (C-3), 175.77 (C-4), 156.09 (C-5), 98.15 (C-6), 163.95 (C-7), 93.29 (C-8), 160.65 (C-9), 102.90 (C-10), 121.90 (C-1′), 115,55 (C-2′), 145.01 (C-3′), 147.66 (C-4′), 115.01 (C-5′), 119.90 (C-6′). The above spectral data were basically consistent with those reported previously [13] and thus, compound **1** was identified as quercetin.

#### Compound 2

A yellow, needle-shaped crystal. Its molecular formula was C_27_H_30_O_15_. EI-MS *m/z*: 594 [M]^+^. ^1^H-NMR (400 MHz, DMSO-*d*_6_) *δ*: 12.57 (1H, s, 5-OH), 10.86 (1H, s, 7-OH), 10.14 (1H, s, 4′-OH), 8.00 (2H, d, *J *= 12.0 Hz, H-2′, 6′), 6.89 (2H, d, *J *= 8.0 Hz, H-3′, 5′), 6.42 (1H, d, *J *= 4.0 Hz, H-8), 6.21 (1H, d, *J *= 4.0 Hz, H-6), 5.32 (1H, d, *J *= 8.0 Hz, H-1′′), 4.44 (1H, brs, H-1′′′); ^13^C-NMR (100 MHz, DMSO-*d*_6_) *δ*: 156.63 (C-2), 133.26 (C-3), 177.43 (C-4), 161.24 (C-5), 98.76 (C-6), 164.14 (C-7), 93.79 (C-8), 156.90 (C-9), 104.04 (C-10), 120.93 (C-1′), 130.93 (C-2′, 6′), 159.93 (C-4′), 115.14 (C-3′, 5′), 101.36 (C-1′′), 74.21 (C-2′′), 76.39 (C-3′′), 69.96 (C-4′′), 75.78 (C-5′′), 66.92 (C-6′′), 100.81 (C-1′′′), 70.39 (C-2′′′), 70.63 (C-3′′′), 71.85 (C-4′′′), 68.29 (C-5′′′), 17.77 (C-6′′′). The above data were basically consistent with those reported in the Ref. [14]. Thus, compound **2** was identified as kaempferol-3-*O*-*α*-l-rhamnosyl-(1 → 6)-*β*-d-glucoside (kaempferol- rutinose).

#### Compound 3

A white powder. The molecular formula was C_8_H_10_O_2_. EI-MS *m/z*: 138 [M]^+^. ^1^H-NMR (C_5_D_5_N, 400 MHz) *δ*: 7.33 (2H, d, *J *= 8.0 Hz, H-2, 6), 7.18 (2H, d, *J *= 8.0 Hz, H-3, 5), 4.11 (2H, d, *J *= 12.0 Hz, H-8), 3.05 (2H, t, *J *= 7.0 Hz, H-7); ^13^C-NMR (C_5_D_5_N, 100 MHz) *δ*: 130.59 (C-1), 130.54 (C-2, 6), 116.07 (C-3, 5), 157.17 (C-4), 39.58 (C-7), 63.86 (C-8). The above data were basically consistent with those reported in the Ref. [15]. Thus, compound **3** was identified as 4-Hydroxyphenethyl alcohol.

#### Compound 4

A white powder. The molecular formula was C_8_H_8_O_4_. EI-MS *m/z*: 168 [M]^+^. ^1^H-NMR (C_5_D_5_N, 400 MHz) *δ*: 8.19 (1H, dd, *J *= 8.0 Hz, 4.0 Hz, H-6), 8.09 (1H, d, *J *= 4.0 Hz, H-2), 7.32 (1H, d, *J *= 8.0 Hz, H-5), 3.74 (3H, s, 3-OCH_3_); ^13^C-NMR (C_5_D_5_N, 100 MHz) *δ*: 123.34 (C-1), 116.03 (C-2), 148.15 (C-3), 152.58 (C-4), 113.61 (C-5), 124.75 (C-6), 168.98 (C-7), 5.58 (C-8). The above data were basically consistent with those reported in the Ref. [16]. Thus, compound **4** was identified as vanillic acid.

#### Compound 5

This compound was a yellow-brown, needle-shaped crystal. The molecular formula was determined to be C_9_H_8_O_4_. EI-MS *m/z*: 180 [M]^+^. ^1^H-NMR (DMSO-*d*_6_, 400 MHz) *δ*: 7.42 (1H, d, *J *= 16.0 Hz, H-7), 7.01 (1H, s, H-2), 6.96 (1H, dd, *J *= 4.0, 8.0 Hz H-6), 6.76 (1H, d, *J *= 8.0 Hz, H-5), 6.18 (1H, d, *J *= 16.0 Hz, H-8); ^13^C-NMR (DMSO-*d*_6_, 100 MHz) *δ*: 125.72 (C-1), 114.60 (C-2), 144.34 (C-3), 148.09 (C-4), 115.36 (C-5), 115.74 (C-6), 145.55 (C-7), 121.04 (C-8), 168.00 (9-COOH). The above data were basically consistent with those reported in the Ref. [17]. Thus, the compound **5** was identified as caffeic acid.

#### Compound 6

A white powder. The molecular formula was C_17_H_24_O_8_. EI-MS *m/z*: 566 [M]^+^. ^1^H-NMR (400 MHz, C_5_D_5_N) *δ*: 6.60 (2H, s, H-3, 5), 6.03 (1H, m, H-*β*), 5.11 (2H, m, H-*γ*), 3.74 (6H, s, 2 × OCH_3_), 3.92–4.38 (6H, m, H-2′–6′), 3.34 (2H, d, *J *= 8.0 Hz, H-*α*); ^13^C-NMR (100 MHz, C_5_D_5_N) *δ*: 153.67 (C-2, 6), 137.82 (C-*β*), 136.26 (C-1), 134.45 (C-4), 115.87 (C-*γ*), 107.13 (C-3, 5), 105.02 (C-1′), 75.98 (C-2′), 78.58 (C-3′), 71.51 (C-4′), 78.28 (C-5′), 62.53 (C-6′), 56.46 (2 × OCH_3_), 40.44 (C-*α*). The above data were basically consistent with those reported in the Ref. [18]. Thus, the compound **6** was identified as dictamnoside A.

#### Compound 7

A yellow powder. The molecular formula was C_24_H_30_O_13_. EI-MS *m/z*: 526 [M]^+^. ^1^H-NMR (400 MHz, C_5_D_5_N) *δ*: 7.52 (1H, s, H-3), 6.65 (1H, s, H-5′′), 6.63 (1H, d, *J *= 1.5 Hz, H-2′′), 6.49 (1H, dd, *J *= 1.5 Hz, 8.5 Hz, H-6′′), 4.18 (2H, m, 2 × H-α), 3.02 (1H, m, H-5), 2.72 (2H, t, *J *= 6.5 Hz 2 × H-β), 2.07 (1H, q, H-9), 4.56 (1H, d, *J *= 8.0 Hz, H-1′), 1.41 (3H, d, *J *= 4.0 Hz, 10-Me); ^13^C-NMR (100 MHz, C_5_D_5_N) *δ*: 94.84 (C-1), 152.92 (C-3), 107.77 (C-4), 26.82 (C-5), 33.53 (C-6), 171.67 (C-7), 73.49 (C-8), 21.26 (C-10), 166.63 (C-11), 99.23 (C-1′), 73.20 (C-2′), 77.34 (C-3′), 70.16 (C-4′), 76.57 (C-5′), 61.38(C-6′), 128.75 (C-1′′), 116.21 (C-2′′), 145.12 (C-3′′), 143.76 (C-4′′), 115.55 (C-5′′), 119.62 (C-6′′), 64.93 (C-*α*), 33.88 (C-*β*). The above data were basically consistent with those reported in the Ref. [19]. Thus, the compound **7** was identified as Lilacoside.

#### Compound 8

A yellow powder. The molecular formula was C_21_H_20_O_12_. EI-MS *m/z*: 465 [M]^+^. ^1^H-NMR (400 MHz, DMSO-*d*_6_) *δ*: 12.65 (1H, s,5-OH), 7.59 (1H, d, *J *= 4.0 Hz, H-6′), 7.57 (1H, d, *J *= 4.0 Hz, H-2′), 6.85 (1H, d, *J *= 8.0 Hz, H-5′), 6.39 (1H, s, H-8), 6.20 (1H, d, *J *= 4.0 Hz, H-6), 5.46 (1H, d, *J* = 8.0 Hz, Glc-H-1′′), 3.17–3.24 (5H, m, Rha-H-2″–6″); ^13^C-NMR (100 MHz, DMSO-*d*_6_) *δ*: 156.11 (C-2), 133.30 (C-3), 177.36 (C-4), 161.18 (C-5), 98.63(C-6), 164.21(C-7), 93.45(C-8), 156.28 (C-9), 103.78 (C-10), 121.52 (C-1′), 115.15 (C-2′), 144.75(C-3′), 148.41(C-4′), 116.16(C-5′), 121.11(C-6′), Glc: 100.92(C-1″), 74.06(C-2″), 77.46(C-3″), 69.92(C-4″), 76.48 (C-5″), 60.95 (C-6″). The above data were basically consistent with those reported in the Ref. [20]. Thus, the compound **8** was identified as quercetin-3-*O*-*β*-d-glucoside.

#### Compound 9

Was a yellow powder. The molecular formula was C_27_H_30_O_16_. EI-MS *m/z*: 610 [M]^+^. ^1^H-NMR (400 MHz, DMSO-*d*_6_) *δ*: 12.60 (1H, s, 5-OH), 10.84 (1H, s, 7-OH), 9.68 (1H, s, 4′-OH), 9.19 (1H, s, 3′-OH), 7.55 (2H, d, *J *= 8.0 Hz, H-2′, 6′), 6.85 (1H, d, *J *= 8.0 Hz, H-5′), 6.39 (1H, d, *J *= 4.0 Hz, H-8), 6.20 (1H, d, *J *= 4.0 Hz, H-6), 5.35 (1H, d, *J *= 4.0 Hz, H-1′′), 4.38 (1H, brs, H-1′′′); ^13^C-NMR (100 MHz, DMSO-*d*_6_) *δ*: 156.42 (C-2), 133.30 (C-3), 177.37 (C-4), 161.23 (C-5), 98.68 (C-6), 164.07 (C-7), 93.59 (C-8), 156.61 (C-9), 103.98 (C-10), 121.18 (C-1′), 115.23 (C-2′), 144.76 (C-3′), 148.42 (C-4′), 116.26 (C-5′), 121.59 (C-6′), 101.17 (C-1′′), 74.08 (C-2′′), 76.44 (C-3′′), 70.00 (C-4′′), 75.91 (C-5′′), 67.01 (C-6′′), 100.76 (C-1′′′), 70.38 (C-2′′′), 70.56 (C-3′′′), 71.84 (C-4′′′), 68.26 (C-5′′′), 17.77 (C-6′′′). The above data were basically consistent with those reported in the Ref. [21]. Thus, the compound **9** was identified as rutin.

#### Compound 10

A white solid. The molecular formula was C_16_H_32_O_2_. EI-MS *m/z*: 256 [M]^+^. ^1^H-NMR (CDCl_3_, 400 MHz) *δ*: 0.88 (3H, t, *J *= 9.0 Hz, H-16), 1.23–1.29 (24H, m, H-4–15), 1.61 (2H, m, H-3), 2.34 (2H, t, *J *= 7.5 Hz, H-2); ^13^C-NMR(CDCl_3_, 100 MHz) *δ*: 179.3 (–COOH), 34.23 (C-2), 24.83 (C-3), 29.21–29.85 (C-4–13), 32.08 (C-14), 22.85 (C-15), 14.27 (C-16). The above data were basically consistent with those reported in the Ref. [22]. Thus, the compound **10** was identified as palmitic acid.

#### Compound 11

A white solid. The molecular formula was C_12_H_24_O_2_. EI-MS *m/z*: 200 [M]^+^. ^1^H-NMR (CDCl_3_, 400 MHz) *δ*: 0.87 (3H, t, *J *= 7.0 Hz, H-12), 1.26 (16H, m, H-4–11), 1.80 (2H, m, H-3), 2.52 (2H, t, *J *= 8.0 Hz, H-2); ^13^C-NMR (CDCl_3_, 100 MHz) *δ*: 175.9(-COOH), 34.89 (C-2), 25.67 (C-3), 29.63–29.99 (C-4–9), 32.14 (C-10), 22.96 (C-11), 14.29 (C-12). The above data were basically consistent with those reported in the Ref. [23]. Thus, the compound **11** was identified as lauric acid.

#### Compound 12

A white powder. The molecular formula was C_30_H_48_O_3_. EI-MS *m/z*: 456 [M]^+^. ^1^H-NMR (C_5_D_5_N, 400 MHz) *δ*: 5.50 (1H, brs, H-12), 3.45 (1H, dd, *J *= 8.0 Hz, 4.0 Hz, H-3), 3.32 (1H, dd, *J *= 4.0 Hz, 4.0 Hz, H-18), 1.28 (3H, s, H-27), 1.24 (3H, s, H-25), 1.02 (3H, s, H-30), 1.01 (3H, s, H-29), 0.95 (3H, s, H-23), 0.89 (3H, s, H-26); ^13^C-NMR (C_5_D_5_N, 100 MHz) *δ*: 38.93 (C-1), 28.09 (C-2), 78.06 (C-3), 39.38 (C-4), 55.80 (C-5), 18.79 (C-6), 39.74 (C-8), 48.11 (C-9), 37.37 (C-10), 23.82 (C-11), 122.54 (C-12), 144.81 (C-13), 42.16 (C-14), 28.31 (C-15), 23.69 (C-16), 46.47 (C-17), 42.00 (C-18), 346.66 (C-19), 30.96 (C-20), 4.21 (C-21), 33.18 (C-22), 28.78 (C-23), 16.55 (C-24), 15.55 (C-25), 17.43 (C-26), 26.17 (C-27), 180.16 (C-28), 233.27 (C-7, 29), 3.76 (C-30). The above data were basically consistent with those reported in the Ref. [16]. Thus, the compound **12** was identified as oleanolic acid.

#### Compound 13

Was a white powder. The molecular formula was C_30_H_48_O_3_. EI-MS *m/z*: 456 [M]^+^. ^1^H-NMR (C_5_D_5_N, 400 MHz) *δ*: 5.49 (1H, s, H-12), 3.46 (1H, dd, *J *= 8.0 Hz, 8.0 Hz, H-3), 2.65 (1H, d, *J *= 8.0 Hz, H-18), 1.25 (3H, s, H-27), 1.23 (3H, s, H-26), 1.05 (3H, s, H-23), 0.96 (3H, d, *J *= 8.0 Hz, H-29), 0.88 (3H, s, H-24), 1.01 (3H, d, *J *= 8.0 Hz, H-30), 1.02 (3H, s, H-25); ^13^C-NMR (C_5_D_5_N, 100 MHz) *δ*: 37.43 (C-1), 28.11 (C-2), 78.09 (C-3), 39.06 (C-4), 55.80 (C-5), 18.77 (C-6), 33.56 (C-7), 39.94 (C-8), 48.02 (C-9), 39.47 (C-10), 23.90 (C-11), 125.63 (C-12), 139.24 (C-13), 42.48 (C-14), 28.80 (C-15), 24.89 (C-16), 48.02 (C-17), 53.52 (C-18), 39.39 (C-19), 39.37 (C-20), 31.06 (C-21), 37.26 (C-22), 28.67 (C-23), 15.67 (C-24), 16.58 (C-25), 17.52 (C-26), 23.61 (C-27), 179.88 (C-28), 17.43 (C-29), 21.42 (C-30). The above data were basically consistent with those reported in the Ref. [16]. Thus, the compound **13** was identified as ursolic acid.

#### Compound 14

A yellow powder. The molecular formula was C_15_H_12_O_5_ EI-MS *m/z*: 272 [M]^+^. ^1^H-NMR (C_5_D_5_N, 400 MHz) *δ*: 12.83 (1H, s, 5-OH), 7.55 (2H, d, *J *= 8.0 Hz, H-2′, 6′), 7.22 (2H, d, *J *= 8.0 Hz H-3′, 5′), 6.49 (1H, d, *J *= 4.0 Hz, H-8), 6.18 (1H, s,H-6), 5.51 (1H, dd, *J *= 4.0 Hz, 4.0 Hz, H-2), 5.32 (2H, s, H-6, 8), 3.33 (1H, dd, *J *= 12.0 Hz, 12.0 Hz, H-3a), 2.90 (1H, dd, *J *= 0 Hz, 4.0 Hz, H-3b); ^13^C-NMR (C_5_D_5_N, 100 MHz) *δ*: 79.65 (C-2), 43.29 (C-3), 196.53 (C-4), 165.16 (C-5), 97.22 (C-6), 168.56 (C-7), 96.11 (C-8), 164.03 (C-9), 102.87 (C-10), 129.76 (C-1′), 128.86 (C-2′, 6′), 116.42 (C-3′, 5′), 159.53 (C-4′). The above data were basically consistent with those reported in the Ref. [24]. Thus, the compound **14** was identified as naringenin.

#### Compound 15

A white powder. The molecular formula was C_35_H_60_O_6_. EI-MS *m/z*: 578[M]^+^. It was compared with reference substance of *β*-daucosterol, no difference was seen between them in term of the TLC detection. Thus compound **15** was identified as *β*-daucosterol.

### Coagulation time test in vitro

**Fig. 2:**
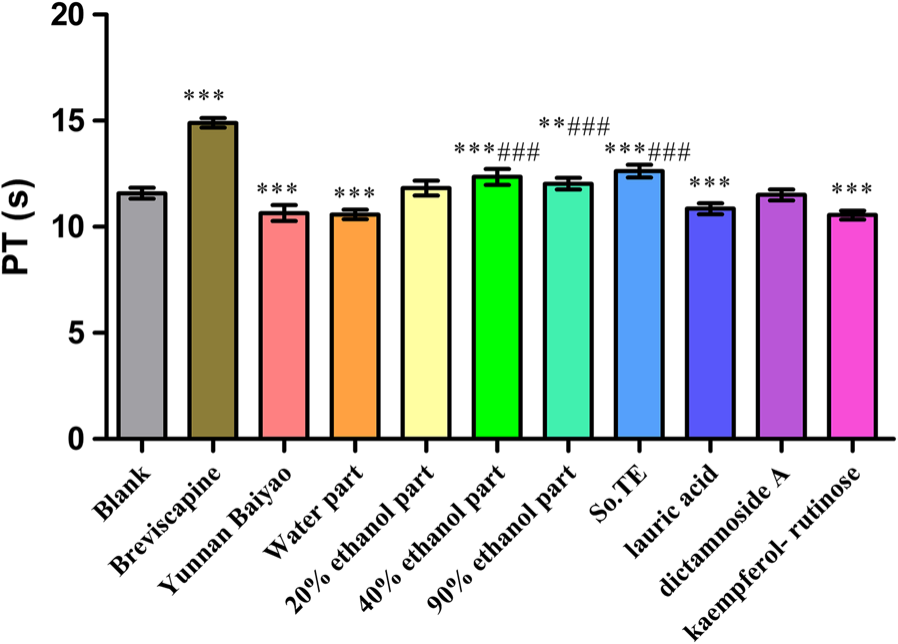
The effects of *S. oblata* extract and compounds on PT in vitro

In Fig. 2, water part, lauric acid and kaempferol-rutinose could significantly shorten PT (*P *< 0.001) compared with the blank group. The 40% ethanol part, 90% ethanol part and So.TE had significant anticoagulant activity (*P *< 0.001 and 0.001 < *P*<0.01) compared with the blank group. The effects of water part, lauric acid and kaempferol-rutinose were not different with that of Yunnan Baiyao.

**Fig. 3:**
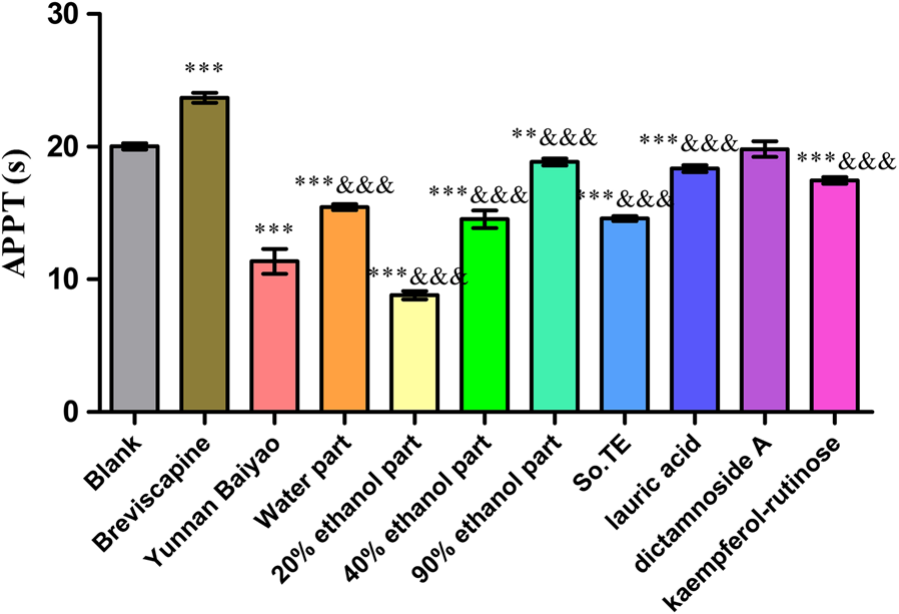
The effects of *S. oblata* extract and compounds on APTT in vitro

In the Fig. 3, all the samples except 60% ethanol part and dictamnoside A could significantly shorten TT (*P *< 0.001 and 0.001 < *P* < 0.01) compared with the blank group. The procoagulant activity of 20% ethanol part was the best one (*P *< 0.001) compared with the Yunnan Baiyao.

**Fig. 4:**
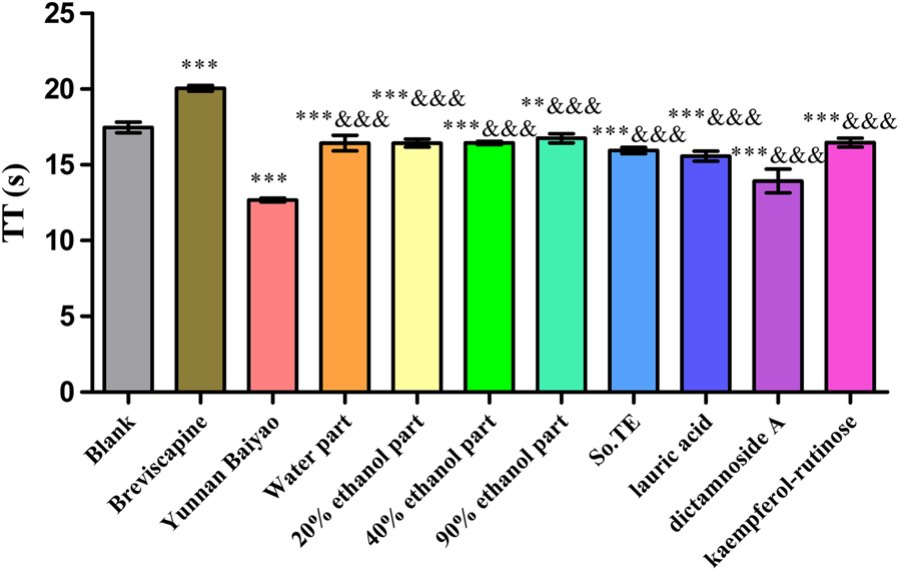
The effects of *S. oblata* extract and compounds on TT in vitro

In Fig. 4, water part, 20% ethanol part, 40% ethanol part, 90% ethanol part, So, TE, lauric acid and kaempferol-rutinose could significantly shorten APTT (*P *< 0.001) compared with the blank group. Water part, 20% ethanol part, 40% ethanol part, 90% ethanol part, So.TE, lauric acid and kaempferol-rutinose had procoagulant activity compared with the Yunnan Baiyao, and 20% ethanol part had a higher activity than that of Yunnan Baiyao, while the others were not better than that of Yunnan Baiyao.

**Fig. 5:**
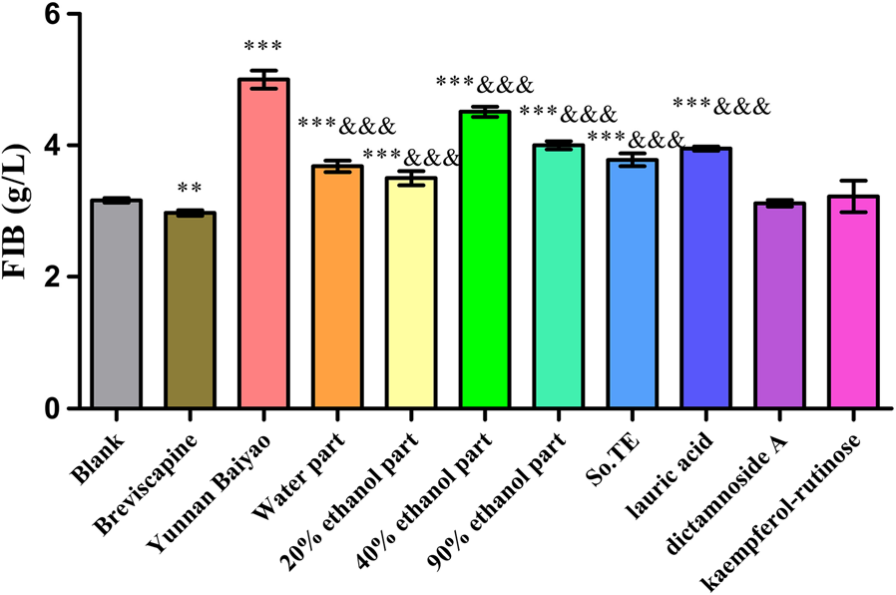
The effects of *S. oblata* extract and compounds on FIB in vitro

In Fig. 5, water part, 20% ethanol part, 40% ethanol part, 90% ethanol part, So.TE and lauric acid all could significantly increase the FIB content (*P *< 0.001) compared with the blank group. The procoagulant activity of the positive control was the best one (*P *< 0.001) compared with the Yunnan Baiyao.

## Discussion

Sun et al. [25] found that the volatile compounds in fresh flowers of *S. oblate* during different flowering periods were different. Dong et al. [6] isolated 8 compounds from the alabastrum of *S. oblate*, and they were identified as syringopicrogenin-B, oleandic acid, ursolic acid, lupanic acid, luprol, *p*-hydroxy phenylpropanol, *p*-hydroxy phenylethanol and *β*-sitosterol. Triterpenic acids were the main components. In this study, fifteen known compounds were isolated from *S. oblata* flowers. They were identified as quercetin (**1)**, kaempferol-3-*O*-*α*-l-rhamnosyl-(1 → 6)-*β*-d-glucoside (**2**, kaempferol-rutinose), 4-Hydroxyphenethyl alcohol (**3**), vanillic acid (**4**), caffeic acid (**5**), dictamnoside A (**6**), Lilacoside (**7**), quercetin-3-O-*β*-D-glucoside (**8**), rutin (**9**), palmitic acid (**10**), lauric acid (**11**), oleanolic acid (**12**), ursolic acid (**13**), naringenin (**14**), and *β*-daucosterol (**15**). Flavonoids, organic acids and Triterpenic acids were the main components.

The previous studies on *S. oblate* flowers were mainly focused on the volatile components and its traditional pharmacological activity. In the present study we found that the *S. oblate* flowers had a significant procoagulant activity for the first time. Our researches showed that water part, lauric acid and kaempferol-rutinose all displayed a significant procoagulant activity, and that the procoagulant activity of water part, lauric acid, and kaempferol-rutinose were not better than that of Yunnan Baiyao, which was used as the positive control.

## Conclusions

In the present study, fifteen compounds were isolated and identified from *S. oblate* flowers, including triterpenic acids, fatty acids and flavonone glycosides etc. Water extract of *S. oblate* flowers, lauric acid and kaempferol-rutinose possessed the procoagulant activity.

## Data Availability

The datasets supporting the conclusions of this article are presented in this main paper. Plant materials used in this study have been identified by Professor Changqin Li. A voucher specimen was deposited in National Center for Research and Development of Edible Fungus Processing Technology.

## Abbreviations

APTT: activated partial thromboplastin time
PT: prothrombin time
TT: thrombin time
FIB: fibrinogen
So.TE: total extract of *S. oblata* flowers
Water part: water extract of *S. oblata* flowers
20% ethanol part: 20% ethanol extract of *S. oblata* flowers
40% ethanol part: 40% ethanol extract of *S. oblata* flowers
60% ethanol part: 60% ethanol extract of *S. oblata* flowers
90% ethanol part: 90% ethanol extract of *S. oblata* flowers

## References

[1] Editorial board of Chinese Materia Medica, State Administration of traditional Chinese medicine (1999). Chinese materia medica (volume eleventh).

[2] Zhao M, Han J, Lv SY, Zhang SJ (2012). Study on chemical constituents in twigs of *Syringa oblate*. Chin Trad Herb Drugs..

[3] Zhang SJ, Guo HQ, Han J, Zhao M, Wang JL (2011). Chemical constituents from seeds of *Syringa oblate*. Chin Trad Herb Drugs..

[4] Zhang SJ, Zhang JF, Wang JL (2006). Chemical constituents in stem bark of *Syringa oblate*. Chin Trad Herb Drugs..

[5] Wang JL, Zhang GF, Dong LW, Zhao M, Zhang SJ (2010). Chemical constituents in seed crust of *Syringa oblate*. Chin Trad Herb Drugs..

[6] Dong LW, Wang JL, Zhao M, Zhang SJ (2011). Chemical constituents of the alabastrum of *Syringa oblata* Lindl. Nat Prod Res Dev..

[7] Tian L, Li YJ, Lv SW, Zhang L, Liu T (2013). Chemical constituents of *Syringa oblate* leaves. Chin J Exp Trad Med Form.

[8] Zhou YM (2005). Studies on the active constituents from leaves of *Syringa oblata* Lindl.

[9] Yang H, Zhao CX, Fang HZ, Wang DS, Zeng YY, Liang YZ (2007). Chemical components in essential oils from *Syringa oblate*. Chin Trad Herb Drugs.

[10] Jiao SQ, Zong MX, Zhang NN, Tao Y (2012). Analysis of the chemical constituents by Supercritical CO_2_ extraction from dried flower of *Syringa oblata* Lindl. Chem Indus For Prod.

[11] Yu TJ, Wang LB, Wu LJ (2016). Research progress of the chemical constituents and pharmacological action of *Syringa* Linn. J Anhui Agric Sci.

[12] Cao PR, Xie PY, Wang XB (2017). Chemical constituents and coagulation activity of *Agastache rugosa*. BMC Complem Altern Med..

[13] Sang YL, Hao YJ, Yang SS (2008). Studies on chemical constituents of *Lamiophlomis rotat*. Chin Trad Herb Drugs..

[14] Yang M (2013). Studies on chemical constituents of persistent calyx of *Physalis pubescens* L.

[15] Zhang YL, Feng ZY, Zheng ZK, Cao YG, Li M, Dong CM (2014). Chemical constituents from the leaves of *Rhemannia glutinosa* Libosch. Chin Pharm J..

[16] Feng WS, Wang JC, He YH, Zheng XK, Song K, Zhang YL (2015). Chemical constituents from the flower buds of *Magnolia biondii* Pamp. Chin Phar J..

[17] Li HB, Yu Y, Wang ZZ, Xiao W, Yao XS (2014). Research on antiviral constituents in Re-Du-Ning Injection (I). Chin Trad Herb Drugs..

[18] Peng W, Han T, Liu QC, Qin LP (2011). Chemical constituents from aerial part of *Atractylodes macrocephala*. China J Chin Materia Med.

[19] Damtoft S, Franzyk H, Jensen SR (1995). Biosynthesis of Iridoids in *Syringa* and *Fraxinus*: carbocyclic iridoid precursors. Phytochemistry.

[20] Ni FY, Chen Z, Xu QM, Yang SL, Chen DF (2013). Chemical constituents from *Rhodiola sachalinensis*. Chin Trad Herb Drugs.

[21] Wen J, Yuan XH, Liu Z (2015). Study on the chemical constituents of *Nerium indicum* Mill. J Anhui Agric Sci.

[22] Liu QR, Li J, Zhao XF, Xu B, Peng WD, Li SX (2016). Studies on constituents from rhizome of *Arundo donax*. Chin Trad Herb Drugs..

[23] Qi J, Shi RF, Yu JM (2016). Chemical constituents from leaves of *Camellia nitidissima* and their potential cytotoxicity on SGC7901 cells. Chin Herb Med.

[24] Zhou XY, Sun XB, Xu XD, Li YD, Zhang HY, Wang YQ (2016). Chemical constituents from seeds of *Caesalpinia sappan*. Chin Trad Herb Drugs.

[25] Sun JW, Yang KY, Li YM, Liu YP (2015). Analysis of aroma components in *Syringa oblate* at different flowering periods by SPME-GC-MS. China Brewing..

